# Revision Total Hip Arthroplasty: Evaluating the Utility of the Geriatric Nutritional Risk Index as a Risk Stratification Tool

**DOI:** 10.1016/j.artd.2024.101430

**Published:** 2024-06-17

**Authors:** Brandon E. Lung, Steven H. Liu, Jane Burgan, Rachel A. Loyst, Amanda Tedesco, James J. Nicholson, William C. McMaster, Steven Yang, Russell Stitzlein

**Affiliations:** a Department of Orthopaedic Surgery, University of California Irvine, Orange, CA, USA; b Renaissance School of Medicine, Stony Brook University, Stony Brook, NY, USA; c School of Medicine, University of California Irvine, Irvine, CA, USA; d Department of Orthopaedic Surgery, Stony Brook University, Stony Brook, NY, USA

**Keywords:** Hip, Revision total hip arthroplasty, Malnutrition, Geriatric, Geriatric nutritional risk index, Postoperative complications

## Abstract

**Background:**

This study investigates the association between the Geriatric Nutritional Risk Index (GNRI), a measure of malnutrition risk, and 30-day postoperative complications following revision total hip arthroplasty (rTHA).

**Methods:**

The American College of Surgeons National Surgical Quality Improvement Program database was queried for all patients ≥65 who underwent aseptic rTHA between 2015 and 2021. The final study population (n = 7119) was divided into 3 groups based on preoperative GNRI: normal/reference (GNRI >98) (n = 4342), moderate malnutrition (92 ≤ GNRI ≤98) (n = 1367), and severe malnutrition (GNRI <92) (n = 1410). Multivariate logistic regression analysis was conducted to investigate the association between preoperative GNRI and 30-day postoperative complications.

**Results:**

After controlling for significant covariates, the risk of experiencing any postoperative complications was significantly higher with both moderate (odds ratio [OR] 2.08, *P* < .001) and severe malnutrition (OR 8.79, *P* < .001). Specifically, moderate malnutrition was independently and significantly associated with deep vein thrombosis (OR 1.01, *P* = .044), blood transfusions (OR 1.78, *P* < .001), nonhome discharge (OR 1.83, *P* < .001), readmission (OR 1.27, *P* = .035), length of stay >2 days (OR 1.98, *P* < .001), and periprosthetic fracture (OR 1.54, *P* = .020). Severe malnutrition was independently and significantly associated with sepsis (OR 3.67, *P* < .001), septic shock (OR 3.75, *P* = .002), pneumonia (OR 2.73, *P* < .001), urinary tract infection (OR 2.04, *P* = .002), deep vein thrombosis (OR 1.01, *P* = .001), pulmonary embolism (OR 2.47, *P* = .019), acute renal failure (OR 8.44, *P* = .011), blood transfusions (OR 2.78, *P* < .001), surgical site infection (OR 2.59, *P* < .001), nonhome discharge (OR 3.36, *P* < .001), readmission (OR 1.69, *P* < .001), unplanned reoperation (OR 1.97, *P* < .001), length of stay >2 days (OR 5.41, *P* < .001), periprosthetic fractures (OR 1.61, *P* = .015), and mortality (OR 2.63, *P* < .001).

**Conclusions:**

Malnutrition has strong predictive value for short-term postoperative complications and has potential as an adjunctive risk stratification tool for geriatric patients undergoing rTHA.

## Introduction

The prevalence of osteoarthritis is rapidly increasing in the United States due to the obesity epidemic and the aging population of the United States [1]. Thus, both younger and older patients are at greater risk of undergoing primary total hip arthroplasty (THA) as well as revision total hip arthroplasty (rTHA), which treats various complications of a prosthetic hip such as aseptic loosening, osteolysis, and instability [2,3]. Current epidemiologic studies predict that the incidence of primary THA in the United States will see an annual increase of approximately 29% [4], with the incidence of rTHA projected to follow a similar trend.

Given the increasing demand for rTHA, understanding the role of patient demographics and conditions perioperatively is crucial. Malnutrition is a well-documented risk factor that is associated with an increased rate of postoperative complications, including increased length of stay (LOS) and mortality, surgical site infections (SSIs), and periprosthetic joint infections following orthopaedic joint replacements [5,6]. While it is understood that nutritional status should be evaluated preoperatively, the role of typically used serum albumin and body mass index (BMI) as measures of malnutrition has been equivocal [5,6].

The Geriatric Nutritional Risk Index (GNRI) is a newer risk index that measures malnutrition in elderly patients by using serum albumin and patients’ ideal body weight [7]. GNRI has been found to be a useful predictor of postoperative complications and more reliable than albumin and BMI in predicting mortality in older patients who undergo emergency surgeries [7,8]. In the context of orthopaedic surgery, GNRI was found to be a strong predictor of adverse postoperative outcomes for total joint arthroplasty [9].

While orthopaedic literature shows that GNRI indicative of malnutrition predicts poorer early postoperative outcomes following primary THA [9], the relationship between GNRI and revision arthroplasty remains unclear. The purpose of this study is to study the prognostic value of preoperative GNRI on postoperative outcomes following rTHA. We hypothesized that patients with malnutrition based on GNRI have a greater risk of early postoperative complications compared to patients with normal GNRI.

## Material and methods

We queried the American College of Surgeons National Surgical Quality Improvement Program (ACS-NSQIP) database for all patients who underwent rTHA between 2015 and 2021. Since the NSQIP database is fully deidentified, this study is exempt from approval by our university’s institutional review board. Data from the NSQIP database is obtained from 600+ hospitals in the United States and is collected by trained surgical clinical reviewers, therefore providing validated 30-day postsurgical outcomes [10].

**Figure 1:**
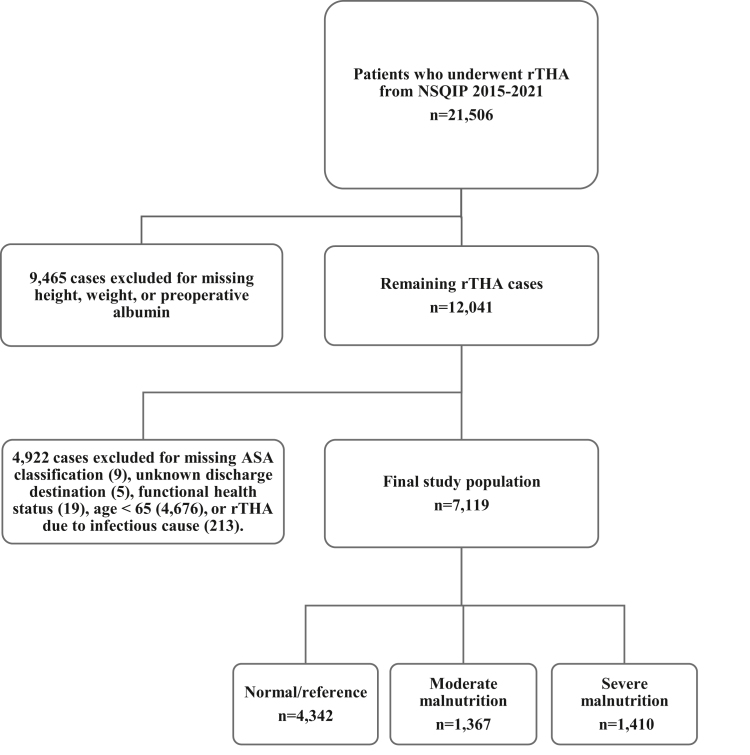
Case selection schematic.

The *Current Procedural Terminology* (CPT) codes corresponding with rTHA (27134—both components, 27137—acetabular component only, 27138—femoral component only) were used to identify patients who underwent rTHA between 2015 and 2021. Revisions secondary to periprosthetic joint infections were excluded because the NSQIP does not provide information related to the chronicity of the infection (acute vs chronic) or the type of revision (one- or two-stage). Therefore, cases with the removal of a prosthesis with or without the insertion of a spacer (CPT codes 27090 and 27091) and either sepsis or septic shock at the time of the operation were excluded from the study. The NSQIP database inherently excludes all cases of patients younger than 18 years of age and all cases with primary admission criteria related to trauma. 21,506 patients met these criteria (Fig. 1). 9465 patients with missing height, weight, or preoperative albumin values required to calculate GNRI were excluded, leaving 12,041 patients. Next, 4922 cases were excluded for missing American Society of Anesthesiologists (ASA) classification, functional health status, age <65, or underwent rTHA due to an infectious cause. GNRI was then calculated for each patient using the following formula, using weight (lb) and albumin (g/L) [[7], [8], [9]]:

$$GNRI=\left(1.489*Albumin\right)+\left(41.7*\frac{Weight}{WLo}\right)$$

WLo represents ideal weight (in kg, then converted to lb for the GNRI calculation), calculated differently based on male or female gender using the Lorentz equations, using height (cm) [[7], [8], [9]]:

$${WLo}_{male}=\left(Height-100\right)+\frac{Height-150}{4}$$

$${WLo}_{female}=\left(Height-100\right)-\frac{Height-150}{2.5}$$

To not miss overweight or obese patients with malnutrition, the ratio of Weight/WLo was capped at 1 for patients whose weight exceeded WLo [8,9].

The remaining study population (Fig. 1) was then indexed into 3 cohorts based on their preoperative GNRI: normal/reference (GNRI >98), moderate malnutrition (92 ≤ GNRI ≤98), and severe malnutrition (GNRI <92). These validated cutoff values for degrees of malnutrition were chosen based on preexisting research on GNRI [9].

Variables collected in this study included patient demographics, comorbidities, surgical characteristics, and 30-day postoperative complication data. Patient demographics included gender, BMI, age, smoking status, functional status, ASA classification, and preoperative steroid use (Table 1). Functional status is classified as “independent” if a patient does not require assistance from another individual for any activities of daily living and “dependent” if a patient requires some or total assistance from another person for activities of daily living. Steroid use status was defined as patients who routinely used immunosuppressants or corticosteroids within 30 days of the preprocedure. Smoking status was defined as cigarette use at any point within the past year before the procedure. Preoperative comorbidities included congestive heart failure (CHF), diabetes, hypertension, severe chronic obstructive pulmonary disease (COPD), bleeding disorders, and disseminated cancer. Thirty-day complications included the following: sepsis, septic shock, pneumonia, unplanned reintubation, urinary tract infection (UTI), cardiac arrest or myocardial infarction, stroke, deep vein thrombosis (DVT), pulmonary embolism (PE), acute renal failure, blood transfusions, on ventilator >48 hours, SSI, wound dehiscence, *Clostridioides difficile* (*C. diff*) infection, nonhome discharge, readmission, unplanned reoperation, LOS >2 days, and mortality.

**Table 1:** Patient demographics and comorbidities for patients with preoperative normal GNRI, moderate malnutrition, and severe malnutrition.

Baseline demographics	Normal (GNRI >98)	Moderate malnutrition (92 ≤ GNRI ≤98)		Severe malnutrition (GNRI <92)	
	Number (%)	Number (%)	*P* value	Number (%)	*P* value
Overall	4342 (100.0)	1367 (100.0)		1410 (100.0)	
Sex			**.023**		.654
Female	2508 (57.8)	837 (61.2)		824 (58.4)	
Male	1834 (42.2)	530 (38.8)		586 (41.6)	
Age			**<.001**		**<.001**
65-69	1211 (27.9)	292 (21.4)		244 (17.3)	
70-79	2155 (49.6)	602 (44.0)		587 (41.6)	
≥80	976 (22.5)	473 (34.6)		579 (41.1)	
BMI (kg/m^2^)			.426		**<.001**
<18.5	60 (1.4)	42 (3.1)		56 (4.0)	
18.5-29.9	2530 (58.3)	786 (57.5)		885 (62.8)	
30-34.9	1051 (24.2)	326 (23.8)		273 (19.4)	
35-39.9	482 (11.1)	131 (9.6)		127 (9.0)	
≥40	219 (5.0)	82 (6.0)		69 (4.9)	
Functional status prior to surgery			**<.001**		**<.001**
Dependent	210 (4.8)	145 (10.6)		333 (23.6)	
Independent	4132 (95.2)	1222 (89.4)		1077 (76.4)	
ASA classification			**<.001**		**<.001**
≤2	1521 (35.0)	290 (21.2)		189 (13.4)	
≥3	2821 (65.0)	1077 (78.8)		1221 (86.6)	
Smoker			**.002**		**<.001**
No	4076 (93.9)	1251 (91.5)		1248 (88.5)	
Yes	266 (6.1)	116 (8.5)		162 (11.5)	
Steroid use			**.025**		**<.001**
No	4071 (93.8)	1258 (92.0)		1274 (90.4)	
Yes	271 (6.2)	109 (8.0)		136 (9.6)	
Comorbidities					
CHF	73 (1.7)	38 (2.8)	**.011**	81 (5.7)	**<.001**
Diabetes	638 (14.7)	222 (16.2)	.164	269 (19.1)	**<.001**
Hypertension	2983 (68.7)	962 (70.4)	.243	998 (70.8)	.142
COPD	228 (5.3)	130 (9.5)	**<.001**	164 (11.6)	**<.001**
Bleeding disorder	200 (4.6)	114 (8.3)	**<.001**	179 (12.7)	**<.001**
Disseminated cancer	28 (0.6)	24 (1.8)	**<.001**	35 (2.5)	**<.001**
Total operation time (minutes)			.657		.342
0-79	667 (15.4)	222 (16.2)		218 (15.5)	
80-128	1517 (34.9)	440 (32.2)		460 (32.6)	
≥129	2157 (49.7)	705 (51.6)		732 (51.9)	

Bold *P* values indicate statistical significance with *P* < .05.

All statistical analyses were conducted using SPSS Software version 26.0 (IBM Corp., Armonk, NY). Patient demographics and comorbidities were compared between cohorts using bivariate logistic regression. Multivariate logistic regression, adjusted for all significantly associated patient demographics and comorbidities for the respective cohort, was used to identify associations between preoperative GNRI and postoperative complications. Odds ratios (ORs) were reported with 95% confidence intervals (CIs). The level of statistical significance was *P* < .05.

## Results

Compared to the normal nutrition group, the moderate malnutrition group was significantly associated with female gender (*P* = .023), older age groups (*P* < .001), dependent functional status (*P* < .001), ASA classification ≥3 (*P* < .001), smokers (*P* = .002), chronic steroid users (*P* = .025), and comorbid CHF (*P* = .011), COPD (*P* < .001), bleeding disorders (*P* < .001), and disseminated cancer (*P* < .001) (Table 2). Compared to the normal nutrition group, the severe malnutrition group was significantly associated with older age groups (*P* < .001), lower BMI groups (*P* < .001), dependent functional status (*P* < .001), ASA classification ≥3 (*P* < .001), smokers (*P* < .001), chronic steroid users (*P* < .001), and comorbid CHF (*P* < .001), diabetes (*P* < .001), COPD (*P* < .001), bleeding disorders (*P* < .001), and disseminated cancer (*P* < .001).

**Table 2:** Bivariate analysis of 30-day postoperative complications in patients with preoperative normal GNRI, moderate malnutrition, and severe malnutrition.

	Normal (GNRI >98)	Moderate malnutrition (92 ≤ GNRI ≤98)		Severe malnutrition (GNRI <92)	
	Number (%)	Number (%)	*P* value	Number (%)	*P* value
Any complication	2653 (61.1)	1101 (80.5)	**<.001**	1347 (95.5)	**<.001**
Medical complications					
Sepsis	23 (0.5)	8 (0.6)	.808	30 (2.1)	**<.001**
Septic shock	10 (0.2)	7 (0.5)	.104	18 (1.3)	**<.001**
Pneumonia	34 (0.8)	16 (1.2)	.183	49 (3.5)	**<.001**
Unplanned reintubation	17 (0.4)	9 (0.7)	.206	11 (0.8)	.074
UTI	50 (1.2)	30 (2.2)	**.005**	45 (3.2)	**<.001**
Cardiac arrest or MI	34 (0.8)	13 (1.0)	.55	24 (1.7)	**.003**
Stroke	6 (0.1)	7 (0.5)	**.018**	2 (0.1)	.974
DVT	138 (3.2)	17 (1.2)	**.019**	28 (2.0)	**<.001**
PE	17 (0.4)	12 (0.9)	**.032**	16 (1.1)	**.002**
Acute renal failure	2 (0.0)	1 (0.1)	.706	7 (0.5)	**.003**
Surgical complication					
Blood transfusions	789 (18.2)	437 (32.0)	**<.001**	640 (45.4)	**<.001**
On ventilator >48 hours	7 (0.2)	4 (0.3)	.341	5 (0.4)	.178
SSI	138 (3.2)	49 (3.6)	.462	125 (8.9)	**<.001**
Wound dehiscence	17 (0.4)	6 (0.4)	.809	9 (0.6)	.235
*Clostridioides difficile* infection	13 (0.3)	2 (0.1)	.345	12 (0.9)	**.009**
Nonhome discharge	1361 (31.3)	704 (51.5)	**<.001**	964 (68.4)	**<.001**
Readmission	308 (7.1)	137 (10.0)	**<.001**	193 (13.7)	**<.001**
Unplanned reoperation	205 (4.7)	90 (6.6)	**.007**	141 (10.0)	**<.001**
Length of stay >2 d	2277 (52.4)	998 (73.0)	**<.001**	1265 (89.7)	**<.001**
Periprosthetic fracture	93 (2.1)	46 (3.4)	**.011**	49 (3.5)	**.005**
Mortality	26 (0.6)	18 (1.3)	**.010**	52 (3.7)	**<.001**

Bold *P* values indicate statistical significance with *P* < .05.

MI, myocardial infarction.

Compared to normal nutrition, the risk of experiencing any postoperative complications was significantly higher with both moderate (*P* < .001) and severe malnutrition (*P* < .001). Specifically, compared to normal nutrition, moderate malnutrition was significantly associated with a greater likelihood of UTI (*P* = .005), stroke (0.018), DVT (*P* = .019), PE (*P* = .032), blood transfusions (*P* < .001), nonhome discharge (*P* < .001), readmission (*P* < .001), unplanned reoperation (*P* = .007), LOS >2 days (*P* < .001), periprosthetic fracture (*P* = .011), and mortality (*P* = .010) (Table 3). Compared to normal nutrition, the severe malnutrition group was significantly associated with a greater likelihood of sepsis (*P* < .001), septic shock (*P* < .001), pneumonia (*P* < .001), UTI (*P* < .001), cardiac arrest or myocardial infarction (*P* = .003), DVT (*P* < .001), PE (*P* = .002), acute renal failure (*P* = .003), blood transfusions (*P* < .001), SSI (*P* < .001), *C. diff* infection (*P* = .009), nonhome discharge (*P* < .001), readmission (*P* < .001), unplanned reoperation (*P* < .001), LOS >2 days (*P* < .001), periprosthetic fracture (*P* = .005), and mortality (*P* < .001).

**Table 3:** Multivariate analysis of 30-day postoperative complications in patients with preoperative normal GNRI, moderate malnutrition, and severe malnutrition.

	Moderate malnutrition (92 ≤ GNRI ≤98)	Severe malnutrition (GNRI <92)
	OR, *P* value (95% CI)	OR, *P* value (95% CI)
Any complication	2.08, **<.001** (1.78-2.42)	8.79, **<.001** (6.74-11.46)
Medical complication		
Sepsis	--	3.67, **<.001** (2.01-6.70)
Septic shock	--	3.75, **.002** (1.62-8.69)
Pneumonia	--	2.73, **<.001** (1.67-4.49)
UTI	1.59, .055 (.99-2.54)	2.04, **.002** (1.31-3.19)
Cardiac arrest or MI	--	1.30, .388 (0.72-2.35)
Stroke	2.99, .055 (0.98-9.19)	--
DVT	1.01, **.044** (1.00-1.01)	1.01, **.001** (1.00-1.01)
PE	1.87, .108 (0.87-3.99)	2.47, **.019** (1.16-5.27)
Acute renal failure	--	8.44, **.011** (1.64-43.52)
Surgical complication		
Blood transfusions	1.78, **<.001** (1.55-2.06)	2.78, **<.001** (2.41-3.20)
SSI	--	2.59, **<.001** (1.96-3.41)
*Clostridioides difficile* infection	--	1.50, .387 (0.60-3.75)
Nonhome discharge	1.83, **<.001** (1.60-2.09)	3.36, **<.001** (2.91-3.87)
Readmission	1.27, **.035** (1.02-1.57)	1.69, **<.001** (1.37-2.09)
Unplanned reoperation	1.29, .056 (0.99-1.69)	1.97, <**.001** (1.54-2.52)
Length of stay >2 d	1.98, **<.001** (1.72-2.28)	5.41, **<.001** (4.48-6.54)
Periprosthetic fracture	1.54, **.020** (1.07-2.23)	1.61, **.015** (1.10-2.35)
Mortality	1.24, .500 (0.66-2.32)	2.63, **<.001** (1.56-4.44)

Dashes represent associations that were not significant in bivariate analysis and were not included in multivariate analysis. Bold *P* values indicate statistical significance with *P* < .05.

MI, myocardial infarction.

All significant patient demographic and comorbidity factors were controlled for using an adjusted multivariate regression analysis: sex, age, BMI, functional status, ASA classification, smoking status, chronic steroid use, and comorbid CHF, diabetes, COPD, bleeding disorders, and disseminated cancer. After controlling for significant covariates, the risk of experiencing any postoperative complications was significantly higher with both moderate (OR 2.08, 95% CI 1.78-2.42; *P* < .001) and severe malnutrition (OR 8.79, 95% CI 6.74-11.46; *P* < .001). Specifically, compared to normal nutrition, moderate malnutrition was independently and significantly associated with DVT (OR 1.01, 95% CI 1.00-1.01; *P* = .044), blood transfusions (OR 1.78, 95% CI 1.55-2.06; *P* < .001), nonhome discharge (OR 1.83, 95% CI 1.60-2.09; *P* < .001), readmission (OR 1.27, 95% CI 1.02-1.57; *P* = .035), LOS >2 days (OR 1.98, 95% CI 1.72-2.28; *P* < .001), and periprosthetic fracture (OR 1.54, 95% CI 1.07-2.23; *P* = .020). Compared to normal nutrition, severe malnutrition was independently and significantly associated with sepsis (OR 3.67, 95% CI 2.01-6.70; *P* < .001), septic shock (OR 3.75, 95% CI 1.62-8.69; *P* = .002), pneumonia (OR 2.73, 95% CI 1.67-4.49; *P* < .001), UTI (OR 2.04, 95% CI 1.31-3.19; *P* = .002), DVT (OR 1.01, 95% CI 1.00-1.01; *P* = .001), PE (OR 2.47, 95% CI 1.16-5.27; *P* = .019), acute renal failure (OR 8.44, 95% CI 1.64-43.52; *P* = .011), blood transfusions (OR 2.78, 95% CI 2.41-3.20; *P* < .001), SSI (OR 2.59, 95% CI 1.96-3.41; *P* < .001), nonhome discharge (OR 3.36, 95% CI 2.91-3.87; *P* < .001), readmission (OR 1.69, 95% CI 1.37-2.09; *P* < .001), unplanned reoperation (OR 1.97, 95% CI 1.54-2.52; *P* < .001), LOS >2 days (OR 5.41, 95% CI 4.48-6.54; *P* < .001), periprosthetic fractures (OR 1.61, 95% CI 1.10-2.35; *P* = .015), and mortality (OR 2.63, 95% CI 1.56-4.44; *P* < .001).

In general, severe malnutrition was independently and significantly associated with a greater number of unique complications compared to moderate malnutrition. In general, for complications common to both moderate and severe malnutrition, severe malnutrition was found to have stronger associations: any complication (OR 2.08 in moderate malnutrition vs 8.79 in severe malnutrition), blood transfusions (OR 1.78 vs 2.78), DVT (OR 1.01 vs 1.01), nonhome discharge (OR 1.83 vs 3.36), readmission (OR 1.27 vs 1.69), LOS >2 days (OR 1.98 vs 5.41), and periprosthetic fracture (OR 1.54 vs 1.61).

## Discussion

Our study suggests that malnutrition plays an important physiological role in determining surgical outcomes. Compared to normal nutrition, moderate malnutrition was independently and significantly associated with a greater likelihood of experiencing any complications, blood transfusions, DVT, nonhome discharge, readmission, LOS >2 days, and periprosthetic fracture. Severe malnutrition was independently and significantly associated with a greater likelihood of experiencing any complication, sepsis, septic shock, pneumonia, UTI, blood transfusions, DVT, PE, acute renal failure, SSI, nonhome discharge, readmission, unplanned reoperation, LOS >2 days, periprosthetic fractures, and mortality. Relative to normal nutrition, severe malnutrition was independently and significantly associated with a greater number of complications and had a stronger association with complications compared to moderate malnutrition.

The relationship between malnutrition and postoperative complications has a well-established physiological basis. Nutritional deficiencies have been shown to lead to a reduced immune response, including decreased phagocyte activity, cytokine production, lymphocyte number, and antibody secretion [6,11]. Consequently, a diminished immune response results in a greater chance of infection, especially in high-stress postsurgical states. Additionally, malnutrition reduces collagen production, which interferes with proper wound healing and prolongs the recovery period [12]. Multiple studies in orthopaedic surgeries have documented increased complications, including disrupted wound healing, muscle weakness, infections, wound drainage, pneumonia, and mortality, that are linked to malnutrition [[12], [13], [14], [15]].

Our analysis found that patients in the moderate and severe malnutrition group were significantly associated with older age, dependent functional status, ASA ≥3, smoking, chronic steroid use, CHF, COPD, bleeding disorder, and disseminated cancer. Patients in the moderate malnutrition group had an additional significant association with female gender, whereas the severe malnutrition group was also associated with lower BMI and diabetes. There is substantial research showing that malnourished patients are more likely to be older female adults and have a greater number of comorbidities [16,17]. The detrimental effects of malnutrition can be exacerbated by other factors associated with poor health outcomes such as smoking and chronic steroid use [[18], [19], [20]]. Altogether, our findings support preexisting literature, which demonstrates that moderate and severely malnourished patients tend to have a greater number of comorbidities.

After controlling for all demographic variables, we found that moderately and severely malnourished patients had a greater likelihood of experiencing any complications. Severely malnourished patients were found to be significantly associated with a greater number of unique complications compared to moderately malnourished patients. This data supports the literature showing that malnutrition is associated with worse postoperative outcomes following orthopaedic surgery. For instance, our findings that malnutrition is associated with several medical complications such as sepsis, septic shock, pneumonia, and UTI agree with several surgery studies that similarly report that GNRI predictive of malnutrition is an independent risk factor for infectious complications [8,9,21]. Our findings that GNRI, indicative of malnutrition, is also associated with surgical complications such as increased length of hospital stay, readmission, and infectious complications support the findings of another study in total joint arthroplasty, which found that patients with lower albumin levels experienced greater rates of longer hospital stays, readmissions, and infections [22].

Given the existing literature indicating a trend of increasing complications associated with worsening malnutrition, it is critical to establish accurate methods of quantifying malnutrition. As the average age of patients who undergo rTHA increases [23], methods of quantifying nutritional status must be able to differentiate between normal changes in aging vs malnutrition. GNRI was developed to quantify the risk of malnutrition in older adults as an alternative to hypoalbuminemia and BMI, which have received criticism as one-dimensional and insufficient in considering the systemic processes related to malnutrition [24]. In our study, only 3.1% and 4.0% of moderately and severely malnourished patients, respectively, had BMI <18.5, showing that the utility of BMI in malnutrition is limited in isolation. However, GNRI accounts for the interaction between body weight and serum albumin, using body weight to modulate the degree of albumin discrepancy required for malnourished classification. For example, patients with an ideal body weight require a greater albumin abnormality to be considered malnourished based on GNRI compared to patients with lower than ideal body weight.

While other malnutrition indices such as the Mini Nutritional Assessment (MNA) have been developed, GNRI has proven to have the most clinical utility. GNRI has been shown to have the best sensitivity in predicting 3- and 6-month mortality rates compared to MNA and was better at predicting outcomes overall [25]. Similarly, GNRI was found to have a higher level of specificity and diagnostic power when compared to MNA [11,26]. Furthermore, GNRI is a simple and efficient method to diagnose malnutrition since it only requires the 3 factors of height, weight, and albumin levels and does not require a caregiver to be present [26]. GNRI is the superior nutritional index in areas including specificity, diagnostic power, and predictability of postoperative complications and should be considered as an adjuvant screening tool for malnutrition in geriatric patients in combination with serum albumin and BMI.

There are limitations in our analysis attributable to the available data in the NSQIP database. First, the postoperative complications were only recorded within a 30-day period, so we are unable to draw conclusions regarding the impact of malnutrition on long-term outcomes. Additionally, there is no data on patient management in terms of preoperative or postoperative nutritional plans, which could impact the malnutrition group in which patients are placed and consequently confound their postoperative complications. Furthermore, the NSQIP database does not report factors such as the experience of the surgeon and the institution type where the procedure was performed. Next, we were limited by the preoperative and intraoperative information available to us. That is, the lack of preoperative diagnosis and intraoperative factors such as blood loss were unable to be accounted for as potential confounders. Finally, since the database was queried for patients based on the presence of corresponding CPT codes, cases were subsequently inseparable by CPT code. Therefore, this study does not account for the possible differences between one- vs two-part revision total knee arthroplasty. These factors may have provided further insight into the results of our study.

This is a novel study that investigates the relationship between GNRI and postoperative outcomes following rTHA. Despite the existing literature on the connection between malnutrition and surgical outcomes, there is no research on the utility of GNRI in the context of outcomes following rTHA. Therefore, this study contributes to the growing research on the negative impact of malnutrition on orthopaedic surgical outcomes. Our results suggest the utility of GNRI in risk-stratifying geriatric patients with poor nutritional status prior to elective rTHA to address modifiable risk factors and promote favorable patient outcomes.

## Conclusions

In geriatric patients with GNRI indicative of malnutrition, the overall rate of complications following rTHA was found to increase with increasing severity of malnutrition. The findings of this study show that GNRI is a strong predictor of early postoperative complications following rTHA in geriatric patients and support its utility as an adjunctive risk stratification tool for geriatric patients undergoing rTHA to help orthopaedic surgeons better select surgical candidates, address modifiable risk factors, and promote favorable patient outcomes.

## Conflicts of interest

W. C. McMaster receives royalties from Innomed. J. J. Nicholson receives research support for diabetes from DEXCOM (not relevant). All other authors declare no potential conflicts of interest.

For full disclosure statements refer to https://doi.org/10.1016/j.artd.2024.101430.

## CRediT authorship contribution statement

**Brandon E. Lung:** Writing – review & editing, Conceptualization. **Steven H. Liu:** Writing – original draft. **Jane Burgan:** Writing – original draft. **Rachel A. Loyst:** Writing – original draft. **Amanda Tedesco:** Writing – review & editing, Project administration. **James J. Nicholson:** Supervision. **William C. McMaster:** Supervision. **Steven Yang:** Supervision. **Russell Stitzlein:** Supervision.

## Appendix A Supplementary Data

Conflict of Interest Statement for Burgan

Conflict of Interest Statement for Liu

Conflict of Interest Statement for Loyst

Conflict of Interest Statement for Lung

Conflict of Interest Statement for McMaster

Conflict of Interest Statement for Nicholson

Conflict of Interest Statement for Stitzlein

Conflict of Interest Statement for Tedesco

Conflict of Interest Statement for Yang
